# Talking About Looking: Three Approaches to Interviewing Carers of People With Rheumatoid Arthritis About Information Seeking

**DOI:** 10.1177/1049732315599373

**Published:** 2015-08-18

**Authors:** Richard Philip Lee, Ben Thompson, Paul Whybrow, Tim Rapley

**Affiliations:** 1 Newcastle University, Newcastle-upon-Tyne, United Kingdom; 2 Newcastle-upon-Tyne Hospitals NHS Foundation Trust, Newcastle-upon-Tyne, United Kingdom

**Keywords:** interviews, health information seeking, musculoskeletal disorders, caregivers / caregiving, technology, qualitative, England

## Abstract

Given the profusion of illness-related information, in this article, we consider how talking about information seeking—and in particular Internet use—is difficult, not because it is necessarily a highly sensitive topic (though it may be), but rather due to the unusual and unfamiliar situation of talking about information seeking. Drawing on interviews conducted as part of a study on the educational needs of carers of people with rheumatoid arthritis, we compare three types of interview for understanding online information seeking: interviews (recall), researcher-led observation (joining participant at the computer), and diaries. We discuss the strengths and weaknesses of each approach and discuss how changing interview questions and the form of interaction can help to produce different types of data, and potentially more meaningful insights. Of the three approaches, conducting interviews with participants while looking at a computer (talking while looking) offered the best opportunities to understand Internet-based information seeking.

## Introduction

The profusion of illness-related information—driven mainly by increased access to the Internet—has given rise to a number of studies concerned with the ways people look for and use information. Valuable insights have been produced into the type of information looked for by patients and the circumstances giving rise to information seeking (Ziebland et al., 2004), Internet use within a broader set of health information practices (Henwood, Wyatt, Hart, & Smith, 2003; Wyatt, Henwood, Hart, & Smith, 2005), approaches to classifying the “trustworthiness” of websites (Nettleton, Burrows & O‘Malley, 2005), and the role of Internet-based information on the experience of illness and the cultivation of expertise (Ziebland, 2004). However, few studies focus, in detail, on the practical action and reasoning undertaken when using the Internet and/or illness-related information.

In this article, we draw on interviews conducted as part of a study that aimed at understanding the educational needs of carers of people with rheumatoid arthritis (RA). By carers, we mean those people, often partners, friends, or other family members, who offer help and support to the person with RA. In this situation, “carers” are involved, in some way, in decisions concerning health and illness. We consider the relationship between the production of qualitative data from interviews, analysis of this data, and understanding how carers of people with RA look for information. We do this to make sense of the way people talk about their information practices as part of a research interaction and to advance our understanding of what and how carers learn about RA. Talking about information seeking—and in particular Internet use—is difficult; not because it is necessarily a highly sensitive topic (though it may be), but rather due to the unusual and unfamiliar situation of talking about (rather than simply undertaking) information seeking. Difficulties exist for the interviewer and the interviewee when talking about looking, often resulting in somewhat generalized accounts that may lack the details of the specific practices used.

Using serial interviews with 10 newly diagnosed patient and carer dyads and single interviews with longer-term review dyads, our aim was to understand these processes as they occur over time. We discuss how changing interview questions and the form of interaction can help to produce different types of data and potentially more meaningful insights. Specifically, we analyze how explicitly orienting some aspects of the interview around a computer enables a different type of discussion. In addition, talking about Internet-based information can give rise to new and relevant interview topics. Despite this, a number of practical issues remain, and we conclude by considering the contribution of different approaches and the implications for understanding information seeking.

### Theories of Information Seeking

Information seeking can be understood as an aspect of education and learning undertaken by people in specific situations, in this case, caring for a person with RA. Whether information seeking is conceptualized primarily as a discrete (cognitive) activity or as an activity embedded within other (social) practices, matters methodologically. For Henwood et al. (2003) and Wyatt et al. (2005), the term “information practices” captures the diverse and everyday activities people undertake to understand their situation. They discuss “information literacy skills,” involving the awareness, retrieval, and discernment of information, and “information landscapes,” which refer to the places people go to for information. In this way, they situate information seeking for health in a social context. Both studies challenge an overly simplified rhetoric of patient empowerment through information. They also draw attention to the ways people resist using the Internet to look for information, whether by assigning responsibility to clinicians, presenting a lack of information literacy skills, or relying on trusted others.

The diverse ways illness-related information is used by people is also considered in an analysis of the concept of “health information-seeking behaviour.” Lambert and Loiselle (2007) recognize the different forms information can take and identify different ways it can be dealt with. They suggest that previous work in the field of communications and information science has moved beyond information seeking to consider information avoidance. For example, Case, Andrews, Johnson, and Allard (2005) suggest that information avoidance has been overlooked due to a preoccupation with the active seeking of information and the information-monitoring actions people undertake. Lambert and Loiselle (2007) propose that anxiety is not always reduced by more information and that the reverse may be true. Although this proposal challenges the characterization of information seeking as, in some way, the only “rational” response to a health-related problem, it does so by offering a dualism of seeking/avoiding information. They conclude that what is needed is an understanding of information seeking as embedded in other social contexts—such as caring for a person with RA—rather than as an isolated process.

The situating of information seeking as one element of information practice is also explored in a review by Harland and Bath (2008). They consider the utility of theories that conceptualize information not as a static repository, but as a part of ongoing sense-making, experience, and belief. They recognize how Dervin’s work on sense-making demands that information be conceptualized as something that is produced through the making (and unmaking) of sense, rather than as a given external entity (Dervin, 1998; Dervin, Foreman-Wernet, & Lauterbach, 2003).

For McKenzie (2003), the use of “information practices” denotes a shift away from an overly cognitive orientation associated with models of information behavior and toward an appreciation of the way information coheres from multiple sources and makes sense in different ways in different situations. Recognizing that information seeking is not an isolated activity but is embedded in everyday life focuses attention on understanding the routines and social contexts in which learning takes place.

Viewing information seeking in this way, Mair and Kierans (2012) take an ethnomethodological approach to investigate patient interactions with information. Looking at interactions around a web-based patient information resource, their findings suggest that attention should be paid to how people read information as part of associated social practices.

Despite the surge of interest at the academic, practitioner, and patient levels, we still understand relatively little about how patients read and link different types of information together for practical purposes in everyday situations. (p. 280)

The “how” of looking and reading is embedded in the everyday use of information technology. As a theory of information seeking, this requires attention to the practical action and reasoning that underpin the identification and interpretation of health-related information.

Our study addressed the finding and use of information by carers. In a review of studies of Internet use by carers of people with cancer, Kinnane and Milne (2010) highlight evidence that both carers and patients prefer advice from health care professionals. They found that carers would value advice on how to focus their use of the Internet, including recommendations for particular websites. In calling for the development of carer-specific applications and websites, Kinnane and Milne address the centrality of search engines (in particular Google™) to the way carers navigate the Internet. The importance of Google™ as a gateway for Internet use has also been recognized by Nettleton et al. (2005) and McTavish, Harris, and Wathen (2011). The latter have considered the implications of the structuring of Google™ search returns, focusing on the content of websites returned on the “first page” of searches related to five different conditions. However, although this approach helps to explore the style, content, and repetition of information from searches, it tells us relatively little about how people interact with Google™ returns as part of their learning practices.

To understand information seeking as a part of information practices, we draw on interviews conducted with people with RA and their nominated informal carer. These interviews dealt, in part, with information seeking conducted using the Internet. As will be discussed, talking about looking for online information is not easy for researchers or participants. The style and format of the interview interaction influences talk, and here we reflect on a series of attempts to change the nature of the research interaction to deepen our understanding of Internet use within everyday information practices.

## Method

The participants in this study were recruited from three hospital-based rheumatology clinics (2 in the North of England, 1 in the East of England) and were identified using purposive sampling and theoretical sampling in line with emerging analysis. The variation sought included the relationship between patient and carer, disease duration, medication history, and age, with theoretical considerations focused on approach to Internet use and information more generally. The first approach was made by a health professional (specialist nurse or consultant rheumatologist), potential participants were issued with patient information leaflets (to inform them of the details of the study), and with consent, all interviews were carried out by the first and third authors in the participants’ homes. Written, informed consent was taken prior to the interview.

Serial interviews were conducted with 11 newly diagnosed dyads (where the patient had been diagnosed with RA within the last 6 months) on three occasions, with an interval of approximately 6 months. In total, 27 interviews were conducted with newly diagnosed dyads; 2 dyads were interviewed twice and 2 dyads once due to interviewees being uncontactable. The person with RA chose the person most involved in their care (the carer), and in the first interview, they were interviewed together. In the second round of interviews, they were given the opportunity to be interviewed together or separately. Where a third interview took place, they were interviewed together. Single interviews were also conducted with 11 review dyads (where the person with RA had been diagnosed for 2 or more years). The characteristics of the newly diagnosed participants and the information-seeking approaches of the carers can be seen in Table 1. The conduct of the interviews—including the use of interview schedules—is discussed fully in the following sections.

**Table 1.** Characteristics of Newly Diagnosed and Review Dyads.

Dyad (Lettering Refers to Recruitment Site)	Person With RA	Time From Diagnosis to First Interview	Relationship of Carer to Person With RA	Carers’ Approach to Condition-Related Information
Newly diagnosed				
Nn1	Female 60s	6 months	Husband 70s	Ongoing use of the Internet to search for RA-related information
Nn2	Female 70s	6 months	Son-in-law (GP) 40s	Ongoing use of professional knowledge and Internet to search for RA-related information
Nn3	Female 60s	2 weeks	Best friend (Female) 60s	Infrequent ongoing use of the Internet to search for RA-related information
Nn4	Female 70s	6 months	Daughter 30s	Has used the Internet to search for RA-related information on a few occasions
Nn5	Male 60s	8 months	Wife 60s	Has sought some additional information beyond consultations
Nn6	Female 20s	6 months	Partner (Male) 20s	Drawn on consultations, Internet, and family member with RA
Nn7	Male 40s	10 months	Wife 40s	Not made aware of husband’s symptoms, looking since diagnosis
Nn8	Female 40s	6 months	Partner (Male) 40s	Primarily learns from partner, but also from doctor
Nn9	Male 50s	4 months	Sister 50s	Has not sought additional information beyond consultations
Sn1	Male 60s	6 months	Wife (former nurse) 60s	Ongoing use of the Internet to search for RA-related information
Sn2	Female 30s	12 months	Husband 30s	Primarily learns from his wife
Review (>2 years from diagnosis of RA)				
Nr1	Male 30s	4 years	Partner 20s	Primarily learns from her partner. Has not used Internet to search for RA information
Nr2	Female 60s	3 years	Sister 60s	Rarely uses Internet to search for RA-related information
Sr1	Female 50s	30 years	Husband 50s	Ongoing use of Internet and printed information
Sr2	Female 60s	35 years	Husband 60s	Frequent use of Internet, leaflets, and other information sources
Sr3	Female 60s	10 years	Son 30s	Rarely uses Internet to search for RA-related information
Ir1	Female 70s	50 years	Husband 70s	Primarily learns from wife. Rarely uses Internet to search for RA information
Ir2	Male 70s	20 years	Wife 60s	Infrequent ongoing use of the Internet to search for medication-related information
Ir3	Female 60s	20 years	Son 50s	Does not use Internet or printed information to learn about RA
Ir4	Female 70s	20 years	Son 40s	Tentative use of Internet searches for RA-related information
Ir5	Female 60s	10 years	Husband 60s	Primarily learns through wife, who searches for information online, though less regularly than in the past
Ir6	Male 70s	6 years	Wife 70s	Relies on information from the hospital and printed leaflets

*Note.* RA = rheumatoid arthritis.

## Findings

Getting people to talk about how they look for information is difficult. People are not used to talking about—and reflecting on—their information practices. Asking participants to describe this activity in an interview setting can be taxing for both the interviewer and the interviewee, although not necessarily through lack of cooperation by interviewee(s) or incompetent interviewing (Roulston, 2014). The focus of this article is on how different interview techniques can be used to enable people to “talk about looking.”

The collaborative work of producing talk requires attention, irrespective of how the data are analyzed (Rapley, 2001). Specifically, this means that what interviewers do and say is important analytically. The presentation and elaboration of interview topics is the primary strategy used for undertaking the interview. A topic is introduced and a question is put to the interviewee. Follow up comments and questions from the interviewer may seek to “unpack” particular aspects of talk during the interview. Eventually, someone (interviewer or interviewee) will shift the topic (though the interviewer may try to shift back). The interactive process produces what Rapley (2001) terms “mentionables”—those things that are introduced into talk and can become a resource for analysis.

The key challenge in this research was how to use serial interviews to understand how carers of people with RA “do” information seeking. Our initial interview schedule to guide the first (joint) interviews with patients and carers dealt with the following topics: circumstances of finding out, current situation, disruptions and anxieties, independent education, and future needs. The interviewer began the interview by asking the person with RA some variation of the following:

So the first thing I wanted to ask you about was when you were diagnosed with the rheumatoid arthritis. Can you remember very much about the circumstances of that?

In the initial joint interviews, the interviewer began the discussion addressing the person with RA, asking them about their diagnosis and inviting them to tell their (RA-related) story. However, the carer seldom remained silent for long, often offering details about diagnosis, health care, and symptoms. In two paired interviews, the person with RA did provide a lengthy, largely uninterrupted narrative of the diagnosis.

Talking about RA experiences requires that the person with the condition be afforded an opportunity to begin to talk about their own experience, even if this quickly takes on the form of a three-way dialogue. Joint interviews of this kind offer scope for a range of styles of interview interaction (Radcliffe, Lowton & Morgan, 2013; Sakellariou, Boniface, & Brown, 2013). Although our intention here is not to develop an analysis of joint interview interactions *per se*, some description of how this was done is helpful in detailing the kind of interactions that took place as part of our experiments in “talking about looking.”

Having established that people nominated as carers are willing to contribute to the illness narrative, further topics and associated questions were introduced to establish how carers go about their information practices, in-keeping with the interview schedule. In most “newly diagnosed” interviews, the carer demonstrated greater involvement in information seeking than the person with RA. The form of this involvement is summarized in Table 1 above. The intention of the research design was to produce detailed accounts of information seeking. Throughout the data collection, we considered how changing the interview interaction might bring about more detailed accounts of information seeking.

We set out three main methods used to develop the interview interaction: discussing information seeking in a typical interview setting, discussing Internet use with the participants while they sat at their computer, and finally having participants complete an Internet diary. The following section discusses each of these approaches in turn.

### Approach 1: Talk to Interviewee(s) About Looking for Condition-Related Information

The first approach used to gather data on participants’ online information seeking was by exploring these in a typical interview setting. During these interviews, the approach was often passive, allowing the participants to talk about their practices and then following up where computer use was mentioned. For example, in the first joint interview with a newly diagnosed dyad, within 2 minutes into the interview, with the talk focusing on the diagnosis, an interviewee (whose wife had been diagnosed with RA 5 months earlier) stated,

Quite often the tests say that you haven’t got it, but you actually have got it, ’cause I read that on the computer.

The patient information leaflet and pre-interview description by the interviewer does detail our focus on the educational needs of carers. Despite this, the significance of the statement is that “the computer” is referred to as a source of evidence to support the prior statement. About 15 minutes later, without information seeking having been raised as a topic, the same interviewee said,

. . . when I started researching into it a little bit and find out what rheumatoid arthritis was er, quite a bit of a nuisance to have [I: Hm], y’know, like erm, not something you just shake off like flu or whatever.

The first mention of computer use related to RA information, followed by this later mention of “researching into it,” provided an opportunity for the interviewer to shift to the topic of carers’ information practices more explicitly. Rather than asking “do you look?” the interviewer directs the question “when you were looking?” toward the carer, directing them to recall details of their information seeking. However, the following excerpt from the same interview demonstrates the difficulty of “talking about looking”:

I: So when you were looking for information then, what is it you were doing? Were you looking at leaflets you’d been given? Or on the computer, or-?

IV: Well mostly on the computer, ’cause I tend to be on me computer at night, and it’s sort of easy to drift on to the topics of the day . . . somebody told me years ago that whatever you’ve got wrong with you, don’t diagnose yourself on, on the Internet . . . you think you’re dying straightaway, you know, erm. But anyway I did get quite a bit of information [I: Hm] from the computer, but I tend to look at the English ones, like the NHS [National Health Service] thing [I: Yeah] ’cause there’s a lot of American ones, an’er they tend to try to flog things more than cure you [I: Hm], y’know. So, . . . so, so I think I’ve got quite a bit of information from there, even though me head’s suddenly emptied of all the information that I’ve got [Interviewees laugh].

The above extract contains a number of themes that were common in the “talking about looking” interviews. Importantly, the participant struggles to recall details of information seeking. The interviewee recognized that he had discriminated between different websites (based on country of origin and commercial intent), but could not recall (under the pressure of the interview situation) the substance of the material he had read. The failure to provide the detailed information is not due to lack of collaboration by the participant or explanation by the interviewer, but rather the difficulty of recalling “looking” as people rarely reflect on such a contextual activity. Similar to the other participants interviewed, the above interviewee’s online learning is not a discrete activity but embedded within the routinized practices of his typical evening. Although these insights are valuable, we felt that different strategies might be needed for follow-up interviews to further our understanding.

First interviews with other newly diagnosed dyads revealed diverse approaches to information, and Internet use was discussed with all. In some cases, the person with RA expressed relatively little desire to search for information, whereas carers had—in varying ways—used the Internet to seek information. In others, carers had spent less time looking for information than the person with RA. Two carers drew on their own clinical expertise as current or former health professionals in combination with Internet use. Although carers were able to provide summaries, or glosses, of how they had searched for information, and the conclusions they drew from these searches, again it proved difficult to generate talk about the process of looking. In the following excerpt from a joint interview, an interviewee (whose best friend had recently been diagnosed with RA) demonstrates this problem:

I: Can you remember where you looked on the Internet? Did you use a search engine like Google or—?

IV: I just put it in on Google you know and different ones came up. There was, y’know like, then about these tablets that you take for the disease like them [methotrexate] you know. Then it was saying you can—once you are stabilized everything can be alright, y’know what I mean? [continues]

The problem here is one of interaction, both in design and in practice. The interviewer asks about locations (“where you looked”) and specifically mentions Google™ as a way to anchor subsequent talk (in the knowledge that this commonly represents the beginning of the looking process online). For the interviewee, the process is not remarkable; she used Google™ and noticed information relevant to the medication her friend had been prescribed (methotrexate) and a positive prognosis. In a second, individual interview with the same interviewee, she was also unable to specify details, though she did situate this within her broader approach to the Internet that moved between believing information, being unsettled by it, and rejecting the Internet as a source of information.

In our second interviews with newly diagnosed dyads, we conducted individual and joint interviews (depending on the preference of the participants) and focused the conversation with carers on their information practices. To do this, three of the authors discussed the potential of introducing interaction around a computer into the interview. The approach is not intended to be naturalistic, unlike eye tracking technology used to measure performance in completing web-based search tasks (Hill, Dickinson, Arnott, Gregor, & McIver, 2011). Instead, it is a means of generating more detailed talk about how carers and patients use Internet-based resources, including the identification and reading of specific websites.

### Approach 2: Talking and Using a Computer

For a second, individual interview with the first interviewee discussed in this section, computer use was moved to a prominent position in the interview schedule. It was decided to focus from the outset on Internet use and therefore introduce the computer into discussion. In developing this schedule, we felt that sitting with the interviewee in front of the computer and working through the things he does and sites he looks at was our best strategy for moving beyond, what we saw as potentially, an overly “surface” discussion. Our expectations were that these specific discussions of his information practices would also produce talk reporting on the ongoing experience of living with his wife’s condition.

IV: . . . [starts to operate mouse] Er, I go on to, like I usually just go here.

I: Yeah, yeah how do you normally, what do you normally do to sort of . . . ?

IV: Like I just, I just go like

[IV typing—8 seconds pause].

I: So, you type rheumatoid arthritis in on Google.

IV: Aye, so obviously it’s coming up, so I’ve done quite a few times.

I: Yeah, different things you look for like treatment or symptoms or diagnosis.

IV: Aye, Wikipedia I don’t do that one.

I: No, why not Wikipedia?

IV: Because it’s just a general description of the thing [I: Yeah]. Whereas, I go on the National NHS, I tend not to go on the American ones [I: Mmm] because they’re; they’re trying to flog stuff. Whereas the, the NHS one . . . [continues]

He goes on to locate his trust in NHS information in terms of an absence of commercial imperative, as in the first interview. From the start of this extract, the interviewee invites the interviewer to observe his “usual” way of doing his Internet searching. He uses Google™, types in “rheumatoid arthritis” and uses the first page of search returns as the contents page with which to navigate. When, as is the case for almost all Internet searches, a Wikipedia entry is displayed on the first page, he volunteers that he does not “do” Wikipedia. He considers Wikipedia as a source of descriptive information that he regards as only offering access to “general” definitional information on RA (“the thing”), reflexively marking such generic definitional information as no longer relevant in his trajectory of information seeking.

Although the interviewee appears to dismiss the value of general descriptive information, he is also not committed to finding information addressing the situation of carers. Later, the interviewer guides the discussion back toward the use of websites by referring to the laptop. The interviewer and interviewee are looking at the Arthritis Research UK website:

I: Yeah. Which is [information] for partners, is there anything, have you ever looked for that sort of information about what can you do to . . . ?

IV: Not specifically but [I: Right] but I’ve come across things like that, [I: Mmm] whilst I’m looking for something else. Do you know what I mean? Sometimes you read things about what, what happens to the people in general [I: Mmm], when you’re not looking for people in general. But you, but you read about it on the way down to get to where you want to be, if you know what I mean?

At the beginning of this interaction, the interviewer broadens the notion of information seeking beyond the etiology and pharmaceutical treatment of RA, to include issues relevant to interviewee’s role as a carer. The interviewer does this by referring to looking for information about “what you can do to . . . ?” The interviewee situates this within his overall account of looking on the Internet, by stating that that sort of information—what you can do to [help]—is not central to his looking (“not specifically”), and instead he comes “across things like that,” which remain unspecified at this stage. He is, therefore, not ignorant of these information sources (and not ignorant that these things might matter) and will look at them in terms of recognizing them and reading them, but they are not part of *his* “looking.”

Having a quest orientation to looking on the Internet—getting to where you want to be—involves the classification of visible material, as identified through concepts such as monitoring (for threats) and blunting (to avoid or distract from threatening information; see Case et al., 2005; Lambert & Loiselle, 2007). The interviewee checks on the “validity” of this, “Do you know what I mean?” before offering a further clarification of what this particular style of looking involves. Classifying material requires existing categories to draw on, in this case, “what happens to the people in general.” The phrase, *the people*, can be heard as those people with RA and their carers—an aggregation of diverse lives and situations. However, the meaning of generality in this passage is less clear. It could refer to a general set of circumstances that arises across all people with RA and their carers (and we note that, in retrospect, here the interviewer could have explored what was meant by general). Particularly striking in this case is the form looking takes when using a computer, mouse, and a web interface. The interviewee states, “but you read about it on the way down to get to where you want to be,” echoing the scrolling action of scanning and reading on a website and giving us an insight into the way looking is performed using a website. In a previous extract, he offers a contrast between the category of “general” information—as the category of information that he is not seeking explicitly at this moment—and his search for “specific” information, “where [he] wants to be,” which motivates his information seeking at this point.

The same method was used in a second interview with an interviewee. Her husband had been diagnosed with RA about a year ago, while she also dealt with her own treatment for breast cancer. She had been advised by MacMillan nurses (charitable sector nurse specialists in cancer care) not to use Internet too much for her own illness. In the extract below, we discuss a link to a news media report of a cure for arthritis (“Single Jab Can Beat Arthritis”), before she makes a comparison with her own condition and mentions her existing knowledge of RA.

IV: . . . That single jab, but I’ve gone on that [I: Mmm] and I, I think that was just a, it, it wasn’t really . . .

I: So that’s one with *The Express* online.

IV: That wouldn’t, I think I’ve gone on that and that wasn’t what I thought it was.

I: Single jab . . .

IV: “Single Jab Can Beat Arthritis” for rheumatoid arthritis. [4 seconds pause while reading] That would be interesting, but then I would probably think, “Well if there was a jab then everybody would have it” y’know? [I: Yeah] So basically, that’s [I: Mmm], I found more information on what was wrong with me than what I did on the arthritis [I: Yeah]. Most of the arthritis ones weren’t, y’know . . . that rheumatoid arthritis diet, I’ve been on that [I: Mmm]. Penn Medicines, I’ve been on that. The symptoms, well I already knew the symptoms, but I think the diet and the medicines, just I was checking, I would go on, you know what I mean? [IV: Yeah] So . . .

I: Would you spend very long going through all the different erm, returns, websites that were pulled up or would you just, go for the first few pages?

IV: I wouldn’t, I don’t tend to go on to these as much [I: Yeah] the next, because to me, the most interesting ones are at the beginning, I don’t know if I’m right [I: Mmm] and the less interesting ones are, you know, [2 seconds pause] or they repeat their selves a lot [I: Mmm], I’ve noticed that.

The interviewer then asked about methods used for identifying websites, which the interviewee discusses in terms of the ranking of “interesting” sites. Noticing the “single jab” article as featured in the U.K. newspaper the *Daily Express* (online edition) occurred through looking at Google returns for “rheumatoid arthritis.” News media items such as this appear in Google returns on publication, and often (but not always) lose their place on the “first page” days later. In this way, the timing of a search can influence the information that is presented. In comparison with another interviewee, this interviewee notices things relevant to RA, but regards much of the information as known or exaggerated (“if there was a jab then everybody would have it”), particularly in comparison with the information she has been seeking in relation to her own illness. The interviewee is looking for things he regards as specific to RA and reads general things while conducting his searching.

Incorporating the computer and Internet use into the interviews facilitated different kinds of talk. The aim was not to recreate exactly the ways people used the Internet but to enable people to ground their discussion of computer use rather than struggle to recall their actions and thoughts in the abstract. In this regard, changing the interaction worked; it enabled the interviewee to describe and explain, in part, their actions by using the computer. It enabled the interviewer to understand more clearly how the interviewee used the Internet and to ask questions based on these understandings.

### Approach 3: Internet Scrapbook and Using a Computer

Although introducing the computer into the interview enabled interactions that moved beyond the abstract recall of websites and searches, we were keen to learn more. In particular, we wanted to discuss the websites interviewees had viewed—which were hard for them to recall in any detail, even when sitting with them with a computer—and for them to reflect on their information practices. Sillence, Briggs, Harris, and Fishwick (2007) made use of log books and diaries to understand participants’ use and perceptions of websites, and we considered a similar approach by using an “Internet scrapbook.” The scrapbook, for which additional ethical approval was granted, is simply a headed electronic document with the stated aim “to provide a means for you to record how you look online for information related to rheumatoid arthritis and living with the condition.”

Of course, this approach only made sense to those dyads where Internet use played a part in their information practices. For one dyad, the person with RA used the Internet more intensively than her partner with regard to condition-relevant information (both were “general” Internet users). She compiled a brief log of her Internet use over a 7-minute period, including the following extract:

Allergic reaction to insect bites. Wanting to take Loratadine (anti-histamine) and checking to see if I am able to take it alongside Methotrexate. I used the following website, using the A-Z function to find M for Methotrexate.

http://www.patient.co.uk/medicine/methotrexate

The webpage wasn’t helpful as it doesn’t mention drug interactions.

Although this participant and her partner were both active users of the Internet, the directly produced data covered only a small amount of activity. The interviewee with RA begins this interview extract by downplaying the information she has recorded, before shifting attention to her partner:

IV1: But that, that’s only, that, this is only one Internet session [I: Right]. I don’t know why I was looking at it at that time of day, erm [slight laugh], but, as far as I gather, unless IV2’s done anything privately—

IV2: I’ve done, done nothing.

IV1: He’s not Goo—You’ve not looked at anything [IV2: No] to do with rheumatoid arthritis since, IV2’s—

IV2: But like I’ve just said though, when [I: Yeah] I found out that she had it I was looking at the time [I: Yeah]. And I, I suppose you learn over the first month or so, and then you kind of don’t look so much.

The second interviewee states that he has “done nothing,” not necessarily as a result of the method, but rather as a result of a change in his information practices over the trajectory of illness. He suggests he has “learned” during an initial phase and since then has been less interested in using the Internet to look up condition-specific information. Following these exchanges, his partner takes up an explanation of what she was doing when she made the entries. She reflects on the issue most concerning her—employment—while recognizing she is “getting on” with life while also attending blood monitoring and consultation appointments.

Asking interviewees—in particular carers—to make use of the scrapbook did not necessarily mean it formed part of a subsequent interview. It was completed only by those that reported making use of the computer. Of those who were approached, some had not been able to open the electronic document (and now described making little use of the Internet for this kind of information seeking) whereas on another occasion, the partner of the person with RA was not well enough to participate in the interview. The interviewee above does not regard the scrapbook as relevant to his ongoing information practices.

## Discussion

Information practices are of significant interest to social scientists engaged in qualitative studies of health. However, identifying and understanding such practices is far from straightforward. In particular, Internet use has become so mundane for many people that articulating Internet-based information practice is difficult. Our reflections on early interview interactions recognized that trying to talk with interviewees about their information practices generated interesting data but did not necessarily capture their practical action and reasoning. We then adapted the interview format and interaction. Table 2 compares the advantages and disadvantages of the three interaction methods we have described.

**Table 2.** Advantages and Disadvantages of Methods of Interview Interaction.

Method	Advantages	Disadvantages
1 Talk to interviewee about looking for condition-related information.	Simplifies interview interaction by concentrating on talk, especially in first interview.	Difficult for interviewee to recall details of information seeking.
2 Talk to interviewee about their use of the computer to look for condition-related information + Do this in front of the computer to explore searches and websites.	Enables interviewee to recall their methods of finding information on the Internet.	Interviewee may be unsure why interviewer is interested in seeing Internet use and whether they are being tested.
	Enables interviewer to see what the interviewee does and to ask emerging questions.	
3 Ask interviewees to complete a diary of Internet use + Talk to interviewees about their diary + Do this in front of the computer to explore and compare searches and websites.	Provides insight into the sources of online information and participant thoughts at that time.	Requires extra work by the interviewees to maintain scrapbook
	Gives a basis for talking about websites visited.	For repeat interviews, care must be taken not to overburden participants, jeopardizing continuing participation.
	Enables both interviewees to recall their methods of finding information on the Internet and to discuss with interviewer and each other.	One interviewee may be less willing to demonstrate and discuss their information seeking.

These changes allowed for modest advances in understanding information practices. Moving from talking about Internet use to talking about Internet use while using a computer gave us a number of new insights. We could get beyond statements of “I just,” “they came up,” or “me head’s just emptied” in reference to looking for information on the Internet, to being able to discuss the practice of looking (including finding sources and judging them). In an example of talking while looking, one interviewee is able to enter into a discussion of how Wikipedia fits into his understanding of relevant information to his wife’s RA (this discussion is also embedded in his previously articulated view on commercial interests and health information). An extract from the second interview takes us further as we understand how he looks across information on the Internet and makes judgments about relevancy “on the way down,” again something that would not be possible without engagement with real content and movement through this content. The concept of serendipity or information encountering has been discussed by Erdelez (1999) and in this respect, our interviewee “bumps” into information about RA, but states his “method” for discriminating between chanced upon information.

By comparison, another interviewee details her approach to “noticing” information, structured by the most “interesting” information being ordered higher in a Google™ search return. In this respect, Google™ can significantly influence the conduct of Internet-based information practices, in keeping with findings of Nettleton et al. (2005) and McTavish et al. (2011). All participants referred to Google™ as the starting point for their “looking.” Talking while looking did require a “set up,” however, and although no participants explicitly queried why the interviewer wanted a demonstration of their Internet use, this meant that the success of the interaction was (potentially) put at risk. For some dyads, Internet-based looking was performed by somebody else (for example, a son-in-law) who did not participate in the interviews.

The use of scrapbooks offered the possibility of discussing information practices that had occurred away from the interview situation, but risked placing a burden on the participants. In their positive appraisal of the role of participant diaries in research, Jacelin and Imperio (2005) recognize that the completion of diaries in their study required the research team to keep in frequent communication with the participants, raising issues of compliance and coercion. Furthermore, where the behavior of interest is routinized and unremarkable to participants, recording such information may be difficult or seem unnecessary, as with our interviewees. However, such scrapbooks can be a valuable resource for interviews, allowing for more detailed or less common insights. The risk of this method of data collection is borne out by the relatively low engagement with the Internet scrapbook as a tool for enabling a third method of interaction. From the final two extracts, we can see how, even for a relatively young couple (late 20s) who do use the Internet frequently (and discuss the Internet having “always been there” through their teenage and adult years), the use of the Internet scrapbook revealed little about the carer’s information practice, as he rarely used the Internet to look for RA-related information. Looking for information was largely an individual activity and involved some delegation of work; one partner was more interested and looked, and then discussed their findings with the other partner.

In this article, we have discussed three approaches to capturing learning practices online, all of which need to contend with the way that online learning, in practice, is embedded within contexts and everyday practices. Such a conception of learning practices corresponds to Dervin’s (1998) notion of “on-going” sense-making and Lambert and Loiselle’s (2007) argument of embedded social contexts. Similarly, McCaughan and McKenna (2007) identify processes of health-related information seeking that are located within the continuous reframing of a person’s understanding and interpretation of information. Of the three interview approaches, conducting interviews with participants while looking at a computer (talking while looking) offered the best opportunity for understanding Internet-based information seeking. It enables the interviewee to recall their methods of finding information on the Internet and enables interviewer to see what the interviewee does and to ask emerging questions. The interviewer and interviewee explore information practices together and the interviewee presents their previous experience of searching—the tacit knowledge of information seeking—in a form that can be witnessed and questioned by the interviewer.

As health researchers, we know that how people learn about illness is of considerable importance to disease management and health policy. However, research into this area consistently highlights the difficulty of separating out such practice for analysis. The challenge of qualitative research into this area is to develop methods that capture the meaningful ways in which participants understand, evaluate, and use learning resources as part of their everyday practices. In this article, we discussed three styles of interview to understand how participants “look” for information: recall in an interview (talking about looking), interviewing involving Internet scrapbooks (records of looking), and interviewing at a computer (looking together). Talking about looking for information through interviewee recall alone often lacked detail and quickly fell into discussing generalities. Using a scrapbook when interviewing provided a much more detailed resource for discussion, but uptake and adherence was poor. Sitting with participants at the computer not only prompted discussion about specific Internet sites but also on the tacit use of computers, such as “scrolling” and “clicking.” Different forms of interview interaction may have more or less relevancy depending on the situations of participants. Further qualitative research into health education may consider using a combination of these approaches, all of which are imperfect ways of tackling the problem of “talking about looking.”

There were some limitations to our study. A relatively small number of interviewees chose to participate in the completion of an Internet scrapbook. We were not able to complete three interviews with each newly diagnosed dyad, limiting our ability to apply the three interview approaches. Also, although the broader study did take account of diverse information sources, in this article, we have not considered routes or pathways of information sources (see, for example, Johnson, Case, Andrews, Allard, & Johnson, 2006).
